# Transitioning to Country Ownership of HIV Programs in Rwanda

**DOI:** 10.1371/journal.pmed.1002075

**Published:** 2016-08-09

**Authors:** Agnes Binagwaho, Ida Kankindi, Eugenie Kayirangwa, Jean Pierre Nyemazi, Sabin Nsanzimana, Fernando Morales, Rose Kadende-Kaiser, Kirstin Woody Scott, Veronicah Mugisha, Ruben Sahabo, Cyprien Baribwira, Leia Isanhart, Anita Asiimwe, Wafaa M. El-Sadr, Pratima L. Raghunathan

**Affiliations:** 1 Rwanda Ministry of Health, Kigali, Rwanda; 2 Harvard Medical School, Boston, Massachusetts, United States of America; 3 Geisel School of Medicine – Dartmouth, Hanover, New Hampshire, United States of America; 4 Division of Global HIV/AIDS, Centers for Disease Control and Prevention, Kigali, Rwanda; 5 CTS Global Services, Los Angeles, California, United States of America; 6 ICAP at Columbia University, Mailman School of Public Health, Columbia University, New York, New York, United States of America; 7 AIDSRelief, Catholic Relief Services, Kigali, Rwanda; 8 Center for Global Health, Centers for Disease Control and Prevention, Atlanta, Georgia, United States of America

## Abstract

Agnes Binagwaho and colleagues describe how Rwanda achieved country ownership of its HIV programs.

**Summary Points**

- Funding from the United States President’s Emergency Plan for AIDS Relief (PEPFAR) program in 2004 significantly bolstered Rwanda’s ability to develop a national HIV program with implementing partners.
- In 2009, after 5 years of expanding HIV services to achieve universal access to antiretroviral treatment, Rwanda and PEPFAR embarked on a sustainability and country ownership phase of the AIDS response.
- Commitment to the following seven principles helped to create a foundation for successful HIV program management transition from implementing nongovernmental partners to management by Rwanda: a political context of integration and decentralization, ownership through national coordination, participation and partnership, equity, efficiency, accountability, and integration of HIV care to strengthen the entire health system.

## Introduction

An objective of development aid is to increase the capacity of recipient countries until they no longer require foreign assistance. Ideally, partners accompany host countries in this development journey by progressively transferring management skills and technical expertise to promote sustainability of country ownership of programs [1,2]. Reviews of donor-funded health programs have highlighted integration into existing government health systems as key to enabling sustainability [3–6]. However, there are few published analyses of how public health programs are transitioned from donor to host country management [2,7].

**Fig 1:**
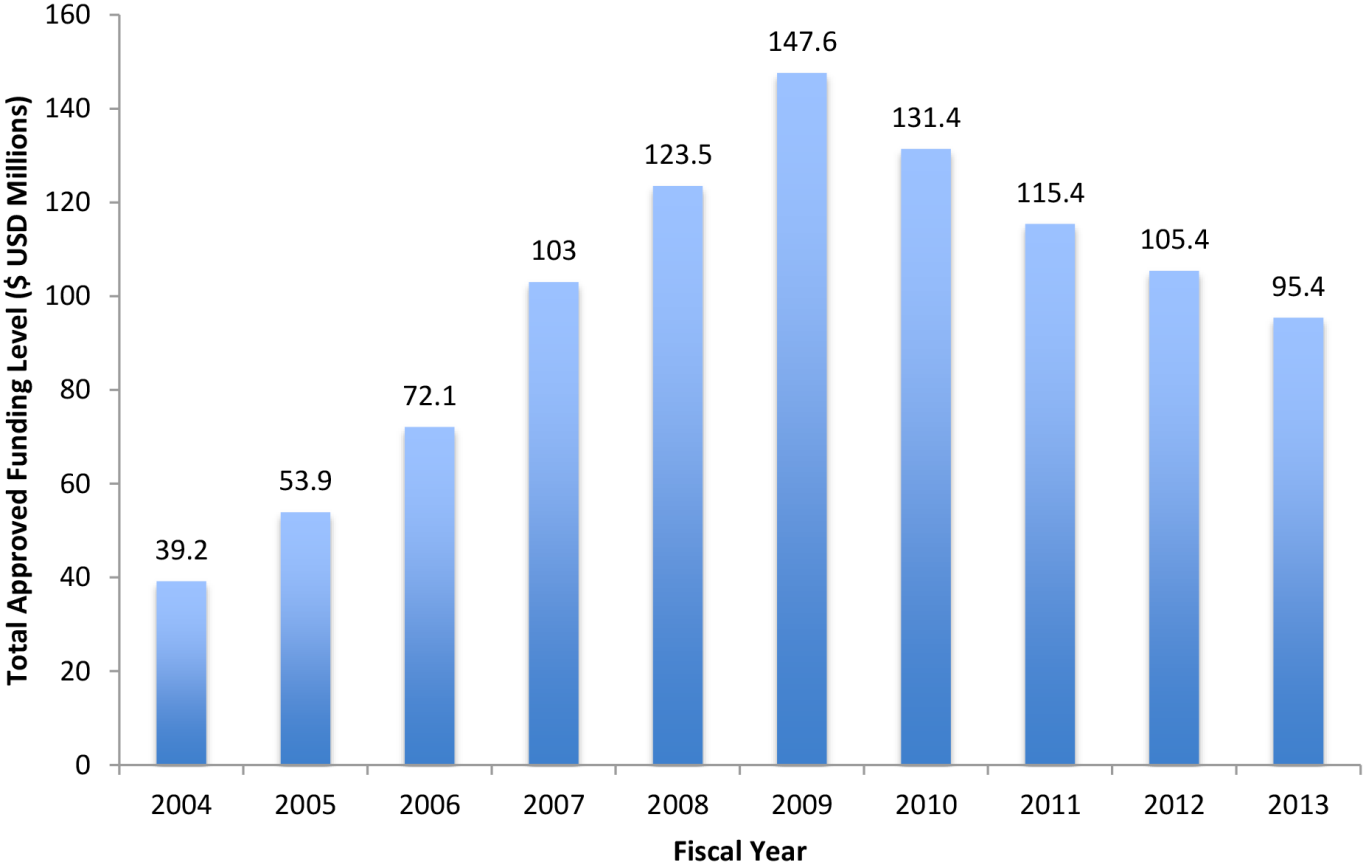
Total PEPFAR approved funding to Rwanda for HIV, tuberculosis (TB)/HIV, and health systems strengthening, 2004–2013. Sources: FY2004–2011: http://www.pepfar.gov/documents/organization/199598.pdf; 2012: http://www.pepfar.gov/documents/organization/212155.pdf; 2013: http://www.pepfar.gov/documents/organization/222179.pdf. FY, Fiscal Year.

As defined through its national policy, *Vision 2020*, Rwanda has committed to reducing its dependence on external aid [8]. In response to the growing AIDS crisis, Rwanda advocated for increasing resources from bilateral, multilateral, and civil society organizations to combat HIV [9,10]. In 2003, the World Bank and the Global Fund for AIDS, Tuberculosis and Malaria (Global Fund) were among the first contributors to the AIDS response in Rwanda [8–10]. The launch of the US President’s Emergency Plan for AIDS Relief (PEPFAR) program in 2004 dramatically increased the funding available to support HIV programs in Rwanda (Fig 1). PEPFAR provided 989.7 million US dollars (USD) from 2004 to 2013, and the Global Fund provided 529.2 million USD from 2003 to 2013 [9–12].

This article describes key principles that allowed for the successful transition of HIV program ownership from the donor-led agencies to Rwanda.

## Launching National Scale-up for HIV Care in Rwanda

Through PEPFAR, the US government (USG) developed partnerships with the Government of Rwanda (GOR) for the national AIDS response. Six US agencies managed PEPFAR funds in Rwanda and supported national HIV scale-up programming; the Centers for Disease Control and Prevention (CDC) oversaw in-country implementation of Rwanda’s national HIV program on behalf of the involved Department of Health and Human Services (HHS) agencies. This partnership helped to scale up a national emergency response to the HIV epidemic. Within 5 years, Rwanda’s national HIV program achieved near-universal access to HIV prevention, care, and treatment (Table 1). Rwanda’s decentralized health sector, as well as its policies that permit for task shifting of services, helped to expeditiously provide universal and comprehensive HIV services throughout the country in a geographically equitable manner.

**Table 1:** Key HIV Program Indicators in Rwanda, 2005–2013.

HIV Program Indicator	2005	2006	2007	2008	2009	2010	2011	2012	2013
Number of individuals who received testing and counseling services for HIV and received their results	687,656	895,324	1,272,848	1,503,503	1,938,507	2,400,731	3,134,423	3,633,647	3,940,775
Percentage of adult population aged 15–49 years who reported having received an HIV test in the last 12 months	(DHS 2005) 11.6% women, 11.0% men					(DHS 2010) 38% women, 37.7% men			
Percentage of pregnant women living with HIV who received ART for PMTCT (%)	56	71	82	84	70^a^	68	79	90	92
Percentage of HIV-infected persons eligible for antiretroviral treatment who received it (%)	45.5	69.1	82.2	65.3^b^	75.8	83.3	89.3	91.5	92.0

Source: Data obtained from Tracnet and the 2005 and 2010 Rwanda Demographic and Health Surveys (DHSs). ART, antiretroviral therapy.

^a^ Reduction from 2008–2009 due to guideline changes and phased implementation of option B, B+ for Prevention of Mother to Child HIV Transmission (PMTCT);

^b^ Reduction from 2007–2008 due to increased number of people in need of ART (denominator) resulting from change of national immunologic eligibility criteria from less than 200 to less than 350 cells/mm^3^ cluster of differentiation 4 (CD4) count.

From the onset, GOR sought to expand its involvement in the direct management of its externally funded health and HIV programs given its commitment to national ownership as articulated in *Vision 2020* [8]. In 2009, Rwanda and its PEPFAR-funded partners aligned to support a second “sustainability and country ownership” phase of the AIDS response, which promoted the transition of HIV clinical service program leadership from partnering nongovernmental organizations (NGOs) to the host country [1,13]. Key within the transition plan developed by Rwanda and its partners was to allow for direct PEPFAR financing to the host country government. The plan also employed existing PEPFAR-funded international NGOs working in Rwanda and experts in HIV program management to accompany GOR as it learned how to best manage PEPFAR-supported programs. By February 2012, program transition occurred: Rwanda’s Ministry of Health (MOH) became a direct recipient of PEPFAR funds and was responsible for coordinating comprehensive HIV services for nearly 40,000 patients in 76 health facilities.

## Prerequisites for Transition: The Foundation for Sustainability

The following seven principles were instrumental in building a foundation that permitted for this successful HIV program leadership transition to occur.

### Political Context of Decentralization

Rwanda’s decentralization policy, implemented in 2005, empowers administrative districts, led by mayors, to coordinate all health activities undertaken within their district health facilities and health-oriented NGOs. Rwanda’s network of 45,000 community health workers (CHWs) is the backbone of the country’s health care system, providing care at the village level. This care then integrates with more advanced care provided at health centers in sectors, district hospitals, and referral hospitals at the central level. The decentralized nature of Rwanda’s health system permitted for effective expansion of HIV services to health facilities in districts. For example, the number of health facilities providing Prevention of Mother to Child HIV Transmission (PMTCT) services increased nationwide from 53 in 2003 to 404 in 2011.

### Ownership through National Coordination

From the beginning, Rwanda endorsed “the three ones” governance principle for its national AIDS response: one national HIV coordinating body, one national HIV strategic plan, and one national monitoring system [14]. NGOs operating in Rwanda with PEPFAR funding were committed to the long-term objectives in the national plan and aligned their HIV service delivery activities accordingly. Harmonizing HIV service delivery approaches across different NGOs early on expedited the eventual consolidation and transfer of these programs to MOH management.

### Participation and Partnership

The national HIV prevention, treatment, and mitigation guidelines were developed through an inclusive, participatory process among key implementation stakeholders (national and international NGOs as well as the beneficiaries). As a result, partners employed the MOH comprehensive HIV treatment protocol, which promoted a common system of procurement and distribution of HIV drugs and consumables. Partners strengthened HIV leadership capacity at MOH through management training, direct skills transfer, and technical support to develop national tools and electronic systems to track HIV program activities at central, health facility, and community levels. This collaboration leveraged synergies across the health sector and accelerated universal access to HIV care and treatment. Further, the sense of solidarity, partnership, and trust between PEPFAR, the international NGOs, and MOH garnered through these experiences was critical for the eventual transition of HIV program leadership from partners to GOR in 2012.

### Equity

Before these partnerships existed to support a more robust HIV response in Rwanda, Rwanda’s MOH worked with civil society to develop an equitable approach for allocating limited antiretroviral therapy (ART) for people living with HIV (PLWH). Each health facility providing ART created an enrollment committee comprised of health professionals, social workers, and representatives of local associations of PLWH to ensure that the most vulnerable had access to treatment. Once there was sufficient treatment for all through PEPFAR funding, this committee was transformed into a therapeutic committee to improve support for PLWH retention in treatment. Moreover, from 2003 onwards, to assure equitable geographic access to treatment, GOR coordinated with its partnering NGOs to assume regional responsibilities. To ensure adequate staffing of these new HIV clinics that were being created to optimize geographic equity to care, a complementary program to build an appropriately trained workforce was developed. This assured the delivery of sophisticated HIV health services in remote areas, facilitating PLWH to be treated near their own communities by Rwandan health professionals under the authority of their own local governmental leaders.

### Efficiency

Operationalizing the geographic mandate of NGO partners reduced implementation costs and promoted efficiency by discouraging duplication and improving coordination. In addition, the national plan required that each NGO partner undertake the full range of HIV services and support supervision, training, and mentorship in an integrated manner. This approach reduced the number of partners and logistics- and transport-related costs, all of which helped to streamline the transition process to host country ownership of the HIV delivery system. In addition, GOR implemented nationally approved salary structures for health personnel in all facilities. By adhering to this salary policy, NGO partners helped to promote staff retention across facilities; this simplified the transition process as health personnel status and salaries remained unaffected once MOH assumed responsibilities for HIV program management in 2012.

### Accountability

Rwanda has implemented a number of transparency, anticorruption, and quality assurance policies for all sectors, including health [15,16]. In 2003, the National AIDS Control Commission created a project management unit to oversee the donor funds for the HIV response directly managed by GOR. However, at the time, Rwandan health institutions had limited financial and administrative capacity to manage the program. Thus, to rapidly implement HIV programs funded by PEPFAR, the USG provided funding directly to international NGOs (rather than directly to GOR) to support the expansion of HIV-related services. Over time, these partnering NGOs helped build the administrative and programmatic capacity of Rwandan institutions at the central, district, health facility, and community levels, with an emphasis on accountability for results.

Rwanda’s HIV program performance improved steadily over the first 5 years of PEPFAR funding (Table 1) [17]. The quality of Rwanda’s HIV program and financial management systems improved over this period, and donor confidence in its administrative and reporting mechanisms was bolstered [16]. Moreover, in 2010, Rwanda began to publicly report semiannual budget execution of domestic and development partners’ projects during the MOH Joint Health Sector Review. These efforts laid the groundwork for the establishment of larger direct cooperative agreements between CDC and Rwandan institutions, which was necessary for successfully transitioning HIV program management from NGO partners to GOR.

### Integration of HIV Care to Strengthen the Health System

Since 2003, Rwandan and international stakeholders have embraced a shared vision that controlling the HIV epidemic requires integration of HIV services within the existing health system. The national policy was to create a sustainable system for Rwandans to manage HIV services for the long term without creating a parallel service delivery system. Partnering NGOs (such as the International Center for AIDS Care and Treatment Programs [ICAP] at Columbia University and AIDSRelief) supported Rwanda’s national policy and applied the chronic disease model to HIV care as a way to strengthen critical components of the Rwandan health system such as infrastructure, personnel, supply chain, clinical management, information systems, and laboratory services [18]. This alignment with Rwanda’s national policy improved integration of HIV patient services and yielded benefits across the entire health system with respect to HIV funds. This allowed Rwanda to build an integrated health service delivery platform, in which HIV could be addressed as a chronic disease.

## Implementing Transition: Overcoming Challenges

Despite the gains made through the effective partnership during the early years of HIV program scale-up, there were several challenges Rwanda encountered when implementing the management transition of PEPFAR-funded HIV programs in 2009.

First, Rwanda’s MOH initially lacked the human resources capacity and technical expertise needed to effectively manage the national scale-up of PEPFAR programs. Despite this limitation, MOH was eager to manage the PEPFAR-funded programs rapidly given the importance of national ownership of critical services delivered in Rwanda. However, during those early years of the emergency response to AIDS, the focus on expanding nationwide access to life-saving care and treatment prevailed over strengthening routine program management capacity. Rwanda could have benefited from taking on more responsibilities for service delivery and reporting even earlier than the transition schedule permitted. This tension between respecting host country ownership and being pragmatic about implementation capacity limitations presented numerous challenges at the onset. Such challenges, however, were overcome through consistent, open communication between GOR and USG partners and the shared vision to optimize access and quality of HIV services provided to all Rwandans in need.

Second, once it became time for implementing NGOs to transition program management to GOR, partners faced an ambitious timeframe to complete this process. Specifically, the transition time mandated by USG meant that between 2009 and 2012, a total of 56,568 HIV-infected patients in care (including 25,206 patients on ART) needed to be transferred to different support systems in a seamless manner and without compromise of the quality of care. To meet this objective, a transition task force was formed, comprised of key Rwanda MOH officials, partner NGOs, and CDC, to both plan and monitor the transition of the 76 health facilities involved with HIV care delivery at the time. A key goal for the task force was to maintain the quality of HIV clinical services throughout the transition, using a jointly agreed timeline with clear performance indicators. The task force conducted readiness, baseline, and follow-up assessments and reviewed program data to identify gaps and areas for improvement. Through reconfiguring staff where needed, meeting regularly with stakeholders, routine visits to health facilities, and improved mentorship and supportive supervision from the central level, the program was transitioned to MOH with sustained clinical and management performance 24 months after transition. This task force illustrates the importance of partners committing to jointly monitoring program performance throughout a transition period. Resource-limited countries are likely to require extensive technical support from partners to reach this decision point. Development partners on the other hand need to be willing to reinforce their support for mentorship, quality improvement, and monitoring and evaluation.

## Looking Ahead

Over the past 10 years, Rwanda has developed the expertise and experience to successfully manage a large cohort of HIV-infected individuals, and its clinicians are now able to provide high-quality care to patients at all stages of HIV disease. When PEPFAR partners like ICAP and AIDSRelief transitioned their responsibilities, some of their experienced staff were directly transferred to Rwanda’s MOH to assist in the mentorship of the program managers and health care providers. However, as scientific knowledge advances and HIV prevention and care evolves, Rwanda may still need more advanced technical assistance to help address HIV transmission hot spots, HIV drug resistance, novel drug regimens, improved diagnostic testing methods, and long-term sequelae of HIV and its treatment. The Rwandan government has contracted with more than 20 American academic institutions with the aim to improve the quality of teaching of Rwandan health professionals, in order to be ready for the upcoming changes in the clinical management and quality improvement of the HIV response and to address other health threats [19]. In addition, as the resources for the global HIV response are plateauing and Rwanda’s health sector is mandated to meet diverse health needs of its population, it is important to further prioritize the available resources towards higher-impact interventions in order to achieve greater results. The continued support of development partners is critical as the country finds its way to self-reliance while building a strong health system.

## Conclusions

A decade ago, HIV was a mortal threat to the Rwandan people and an overwhelming challenge for the Rwandan health system. Today, Rwanda has a robust system for delivering quality care to all those affected by HIV [20]. This was possible through the collaboration between GOR and its partners to develop a program from the onset that would allow for national ownership, sustainable development, and patient-centered health care [19].

Rwanda’s HIV program transition experience illustrates both the importance of building capacity and systems within host countries to manage programs as well as the importance of jointly monitoring results to bolster collaboration. Several prerequisites that enabled the successful HIV program transition in Rwanda were identified, including understanding the political context of integration and decentralization, ownership through national coordination, participation and partnership, equity, efficiency, accountability, and developing HIV delivery systems that simultaneously strengthened the entire health system.

Transition of donor-funded programs to country management is an important step on the development pathway and should be built into any program at the very beginning for more efficiency. To offer life-saving services to their people in a sustainable way, governments should strategize with their partners to achieve program transition of HIV delivery and beyond. Countries should seek more program management responsibility and request—as needed—specialized external technical support that helps to assure access to care, high quality, and accountability for the populations in need.

## Abbreviations

ART: antiretroviral therapy
CD4: cluster of differentiation 4
CDC: Centers for Disease Control and Prevention
CHW: community health worker
DHS: Demographic and Health Survey
FY: Fiscal Year
Global Fund: Global Fund for AIDS, Tuberculosis and Malaria
GOR: Government of Rwanda
HHS: Department of Health and Human Services
ICAP: International Center for AIDS Care and Treatment Programs
MOH: Ministry of Health
NGO: nongovernmental organization
PEPFAR: United States President’s Emergency Plan for AIDS Relief
PLWH: people living with HIV
PMTCT: Prevention of Mother to Child HIV Transmission
TB: tuberculosis
USD: United States dollars
USG: United States government
